# Postoperative Intravenous Drip Infusion is not Required after Minilaparotomy Cholecystectomy

**DOI:** 10.1155/1997/92494

**Published:** 1997

**Authors:** N. Kumar, K. B. Annudath, H. S. Shukla, A. Singh, K. Kumar

**Affiliations:** Department of Surgery Institute of Medical Sciences Banaras Hindu University Varanasi 221005 India

## Abstract

*Objective*: To determine if drip infusion should be discontinued after full recovery of the patient from anaesthesia after minilaparotomy cholecystectomy in uncomplicated cases.

*Design*: A randomised controlled clinical trial on 60 patients, from the waiting list, of cholelithiasis/cholecystitis operated by minilaparotomy cholecystectomy between November 1995 to March 1996. 30 patients did not receive postoperative IV drip infusion and in 30 patients 12–24 hours of standard drip transfusion was continued according to the current practice.

*Setting*: Single Surgical Unit, SS Hospital, Banaras Hindu University, Varanasi, India.

*Main outcome measure*: Recognition of clinical indication for continuation of. IV drip infusion after full recovery from anaesthesia.

*Results*: In the cohorts of 30 patients each who were or were not given IV drip infusion after full recovery from anaesthesia following minilaparotomy cholecystectomy the observations on pulse rate, blood pressure, time to first voiding of urine and time to start first oral intake of fluids were identical. However postoperative urinary retention occured in 6 (20%) patients in whom the IV drip infusion was given.

*Conclusion*: There is no clinical indication to continue IV drip infusion after full recovery from anaesthesia in patients operated for minilaparotomy cholecystectomy.


*HPB Surgery*, 1997, Vol. 10, pp. 279–281
Reprints available directly from the publisher
Photocopying permitted by license only

© 1997 OPA (Overseas Publishers Association)
Amsterdam B. V. Published in The Netherlands
by Harwood Academic Publishers
Printed in India

### Postoperative Intravenous Drip Infusion is not Required after Minilaparotomy Cholecystectomy

N. KUMAR[a], K. B. ANNUDATH[b], H. S. SHUKLA[c], A. SINGH[d] and K. KUMAR[e]

[a]*Junior Resident*, [b]*Junior Resident*, [c]*Professor of Surgery*, [d]*Senior Resident*, [e]*Senior Research Fellow, Department of Surgery, Institute of Medical Sciences, Banaras Hindu University, Varanasi 221005, India*

*(Received 30 May 1996)*

***Objective:*** **To determine if drip infusion should be discontinued after full recovery of the patient from anaesthesia after minilaparotomy cholecystectomy in uncomplicated cases.**
***Design:*** **A randomised controlled clinical trial on 60 patients, from the waiting list, of cholelithiasis/ cholecystitis operated by minilaparotomy cholecystectomy between November 1995 to March 1996. 30 patients did not receive postoperative IV drip infusion and in 30 patients 12–24 hours of standard drip transfusion was continued according to the current practice.**
***Setting:*** **Single Surgical Unit, SS Hospital, Banaras Hindu University, Varanasi, India.**
***Main outcome measure:*** **Recognition of clinical indication for continuation of IV drip infusion after full recovery from anaesthesia.**
***Results:*** **In the cohorts of 30 patients each who were or were not given IV drip infusion after full recovery from anaesthesia following minilaparotomy cholecystectomy the observations on pulse rate, blood pressure, time to first voiding of urine and time to start first oral intake of fluids were identical. However postoperative urinary retention occured in 6 (20%) patients in whom the IV drip infusion was given.**
***Conclusion:*** **There is no clinical indication to continue IV drip infusion after full recovery from anaesthesia in patients operated for minilaparotomy cholecystectomy.**

*Keywords:* Minilaparotomy cholecystectomy, IV drip

### 1. INTRODUCTION

It is a tradition to give IV drip infusion for 12–24 hours after standard cholecystectomy [1]. There is no report in the literature to suggest that in patients operated by minilaparotomy cholecystectomy, where tissue handling is less, the postoperative intravenous drip requirement is different to patients operated by standard method of cholecystectomy. We have carried out this prospective randomised study to examine the postoperative intravenous fluid requirement in patients operated by minilaparotomy cholecystomy.

### 2. METHODS

This study was carried out between November 1995 to March 1996. Patients with cholelithiasis

Address for correspondence: Professor H. S. Shukla, 7 SPG Colony, Lanka, Varanasi 221005, India.

from the waiting list for routine cholecystectomy were prospectively randomised in two groups of study and control patients. The patients in control group were given routine postoperative intravenous drip infusion for 12–24 hours but in the study group intravenous drip infusion was discontinued as soon as the patient recovered from the effects of anaesthesia. Patients in both the group were operated under general anaesthesia by minilaparotomy, 3–6 cm subcostal incision, given the same antibiotic prophylaxis of 1 gm intravenous Cefotaxim with pre-anaesthetic medication, the same analgesic of 15 mg Pentazocine every 6 hours and the same prokinetic agent of Metclopromide every 8 hours. The peritoneum and muscle layers were closed separately with continuous Vicryl ("0") suture and skin with subcuticular prolene. The gall bladder bed was drained on merit with a suction drain. In the postoperative period pulse, blood pressure, first voiding time of urine and any other complaints were recorded.

### 3. RESULTS

This study was completed in 5 months period between November 1995 to March 1996. 30 patients each were entered in study (No postoperative IV drip) and 30 in control group (Routine postoperative IV drip). Table I shows patient characteristics. The age, sex, number and type of gall stones and operative findings were similar in both groups of patients. Radivac drain was used in 2 patients in each group and the size of skin incision varied from 3–6 cms in all patients.

The postoperative observations are shown on Table II. Operating time was 36.9 ± 12.1 minute in study and 38.4 ± 10.1 (NS) in control cases. During operation the type and amount of intravenous fluid infusion was similar in two group of patients, but all in all the study group of patients received 691 ± 22.3 ml and control group of patients 2709 ± 1216 ml ($p<0.001$) intravenous fluids. The total duration of intravenous fluid infusion was 48.6 ± 13.8 minutes for control and 798 ± 50.4 minutes ($p<0.001$) for control group of patients.

TABLE I Patients characteristics

| Observations | Study group (30) | Control group (30) |
|---|---|---|
| Male | 2 | 5 |
| Female | 28 | 25 |
| Age | 37.1±9.6<br>24–58 years | 41.8±13.3<br>25–70 years |
| Number of stones | | |
| None | 2 | 1 |
| Single | 11 | 8 |
| Multiple | 17 | 21 |
| Type of stones | | |
| Cholesterol | 17 | 20 |
| Mixed | 11 | 9 |
| Easy operation | 25 | 24 |
| Difficult operation | 5 | 6 |
| Redivac drain | 2 | 2 |

The observation of vital signs (Tab. II) at the conclusion of operative procedure and at two hourly interval thereafter showed no difference in the two group of patients. The urine was voided at 10 hours postoperative by all patients in study and 24 patients in control group of patients. Distension of urinary bladder occurred in 6 (20%) of control group of patients only. Three of these patients required catheterisation to overcome urinary retention. One patient each in study group developed severe retching not controlled by Metclopromide and low blood pressure of 80/60 mm Hg. In both these patients IV drip infusion was restarted after 4 hours for a duration of 4–6 hours. Both recovered spontaneously. Two patients of control group but none in the study group developed severe superficial thrombophlebitis. Oral fluid intake was allowed after 4–6 hours of surgery in both groups but the patients in study group accepted the advise more readily.

TABLE II Observations

| Observations | Study group (30) | Control Group (30) | P Value |
|---|---|---|---|
| Operating time (Minutes) | 36.9 ± 12.1 | 38.4 ± 10.0 | NS |
| Total IV fluids ml | 691 ± 22.3 | 2709 ± 1216 | < 0.001 |
| Duration of IV drip (Minutes) | 48.6 ± 13.8 | 798 ± 504 | < 0.001 |
| Pulse in OT | 88 ± 12 | 89 ± 14 | NS |
| Pulse 6 hrs post OP | 94 ± 13 | 89 ± 14 | NS |
| BP in OT Systolic (mm Hg) | 125.1 ± 11.1 | 123.4 ± 10.0 | NS |
| BP 6 hrs post OP Systolic (mm Hg) | 126.1 ± 14 | 127 ± 17 | NS |
| Time to void urine (Hours) | 10.3 ± 3.0 | 10.2 ± 2.8 | NS |

### 4. DISCUSSION

Postoperative intravenous drip infusion for 12–24 hours following cholecystectomy is a necessity to supplement body's requirement of 30 ml/kg body weight fluid in 24 hours [2] and electrolytes in the interim period of recovery of gastrointestinal function. Whereas application of this sound principle of surgery is true for patients operated by traditional cholecystectomy, in patients operated through minimal access such as minilaparotomy a 12–24 hours duration of postoperative drip infusion appears excessive. Due to minimal access to the gall bladder there is very little handling of the duodenum and intestines hence disruption of their absorptive function is also minmal. This combined with oral fluid intake within 4 hours after operation removes any need for intravenous fluid administration as shown by this study.

Restriction of intravenous fluid in the postoperative period in uncomplicated patients of minilaparotomy cholecystectomy reduces work load of the nursing staff, saves on the cost of cholecystectomy and it increases the confidence of the patient for early mobilisation and fluid intake. There is also less postoperative morbidity, 6 (20%) patients of routine infusion group developed distension of urniary bladder, 3 requiring catheterisation and in 2 additional patients there was thrombophlebitis related to IV infusion. The results of this study should be seen with respect to inclusion of simple cholecystectomy through minilaparotomy in the sphere of day care surgery. That there was no complication in patients not given postoperative drip infusion confirms the thesis that patients operated for minilaparotomy cholecystectomy do not require postoperative IV fluid infusion. It augurs a welcome sign for the patients operated for minilaparotomy cholecystectomy. Cholecystectomy without the drip matches minimal access for cholecystectomy. The results of this paper should please the patients for yet one more less source of morbidity and the health managers for economy of cholecystectomy.

### *References*

[1] Miller, T. A. and Duke, J. H. Jr. (1983). Fluid and electrolyte management in Manual of Preoperative and Postoperative Care. Edited by Dudrick, S. J., Bane, A. E., Eisman, B., Maclean, L. D., Rowe, M. I. and Sheldon, G. F. Publishers WB Saunders Company Philadelphia 38–67.

[2] Jones, R. S. and Meyars, W. C. (1983). The biliary tract and exocrine pancrease in Manual of Preoperative and Postoperative Care. Edited by Dudrick, S. J., Bane, A. E., Eisman, B., Mac Lean, L. D., Rowe, M. I. and Sheldon, G. F. Publishers WB Saunders Company Philadelphia 443–458.

